# Toll-Like Receptor Induced CD11b and L-Selectin Response in Patients with Coronary Artery Disease

**DOI:** 10.1371/journal.pone.0060467

**Published:** 2013-04-03

**Authors:** Ellen H. A. M. Elsenberg, Marieke A. Hillaert, Hester M. den Ruijter, Jan-Willem E. M. Sels, Vincent P. W. Scholtes, Hendrik M. Nathoe, Johan Kuiper, J. Wouter Jukema, Pieter A. Doevendans, Gerard Pasterkamp, Imo E. Hoefer

**Affiliations:** 1 Laboratory of Experimental Cardiology, University Medical Centre Utrecht, Utrecht, The Netherlands; 2 Department of Cardiology, University Medical Centre Utrecht, Utrecht, The Netherlands; 3 Department of Cardiology, Catharina Hospital Eindhoven, Eindhoven, The Netherlands; 4 Division of Biopharmaceuticals, Leiden University, Leiden, The Netherlands; 5 Department of Cardiology, Leiden University Medical Center, Leiden, The Netherlands; University of Tor Vergata, Italy

## Abstract

Toll-Like Receptor (TLR) -2 and -4 expression and TLR-induced cytokine response of inflammatory cells are related to atherogenesis and atherosclerotic plaque progression. We examined whether immediate TLR induced changes in CD11b and L-selectin (CD62L) expression are able to discriminate the presence and severity of atherosclerotic disease by exploring single dose whole blood TLR stimulation and detailed dose-response curves. Blood samples were obtained from 125 coronary artery disease (CAD) patients and 28 controls. CD11b and L-selectin expression on CD14+ monocytes was measured after whole blood stimulation with multiple concentrations of the TLR4 ligand LPS (0.01–10 ng/ml) and the TLR2 ligand P3C (0.5–500 ng/ml). Subsequently, dose-response curves were created and the following parameters were calculated: hillslope, EC50, area under the curve (AUC) and delta. These parameters provide information about the maximum response following activation, as well as the minimum trigger required to induce activation and the intensity of the response. CAD patients showed a significantly higher L-selectin, but not CD11b response to TLR ligation than controls after single dose stimulations as well as significant differences in the hillslope and EC50 of the dose-response curves. Within the CAD patient group, dose-response curves of L-selectin showed significant differences in the presence of hypertension, dyslipidemia, coronary occlusion and degree of stenosis, whereas CD11b expression had the strongest discriminating power after single dose stimulation. In conclusion, single dose stimulations and dose-response curves of CD11b and L-selectin expression after TLR stimulation provide diverse but limited information about atherosclerotic disease severity in stable angina patients. However, both single dose stimulation and dose-response curves of LPS-induced L-selectin expression can discriminate between controls and CAD patients.

## Introduction

Atherosclerosis is an inflammatory disease in which monocytes and macrophages play an essential role [1]. Toll-like receptors (TLRs), abundantly expressed by most inflammatory cells, are important for the induction of innate immune responses and can be activated by both pathogens and endogenous ligands. TLRs, and especially TLR2 and TLR4, are involved in the initiation and progression of atherosclerotic disease. Multiple studies have shown that TLR2 and TLR4 are up-regulated in atherosclerotic plaques and circulating monocytes and that this enhanced expression is associated with more severe atherosclerotic disease [2]–[5]. Both TLR2 and TLR4 expression as well as the inflammatory response following TLR ligation, differ among individuals and may correlate with clinical presentation. Previous studies demonstrated that isolated monocytes from patients with unstable angina (UA) and acute myocardial infarction (AMI) produce higher levels of B7-1 and Interleukin-12 (IL-12) following LPS stimulation [6] and patients with UA showed an enhanced TLR response of circulating monocytes after whole-blood LPS stimulation, as assessed by IL-6 secretion [7]. Moreover, an increased response to TLR activation seems to be associated with atherosclerotic disease severity which was shown in a cohort of patients with stable angina (SA) [8].

However, comparisons of reported results are cumbersome due to the use of non-standardized protocols with different incubation times and readouts to assess TLR responsiveness. For potential clinical application of TLR responsiveness as a measure of disease severity, standardization is therefore mandatory. In most previous studies the activation status of inflammatory cells following TLR ligation was assessed after stimulation with a single, high concentration of a TLR ligand. However, dose-response curves might differ among individuals not only in terms of maximum activation, but also in the steepness of the response with increasing dosage and in the minimum ligand concentration needed to induce an inflammatory response.

Furthermore, the majority of studies in coronary artery disease (CAD) patients used cytokine release as read-out for TLR response. This usually requires incubations of several hours, which is less favorable for diagnostic testing. Hence, activation markers that respond quickly after TLR activation and for which no *de novo* protein synthesis is needed seem more appropriate for successful future applications.

CD11b and L-selectin (CD62L) are surface activation markers and play a role in the adhesion of inflammatory circulating monocytes and neutrophils, which is an important step in the initiation of atherosclerosis. L-selectin is mainly involved in leukocyte rolling over endothelium while CD11b is responsible for subsequent firm adhesion [9]–[12]. Circulating leukocytes express both markers also under normal circumstances, but expression is quickly changed by inflammatory stimuli. Upon activation, CD11b levels are usually up-regulated while L-selectin is shed from the cell surface releasing an active soluble form in the circulation [13], [14]. Both markers have been associated with atherosclerotic disease in several reports [15]–[17].

In this exploratory study, we assessed whether single dose response and detailed dose-response curves of TLR2 and -4 induced CD11b and L-selectin expression differs between patients and controls. Furthermore, we explored the potential diagnostic and prognostic value of TLR response by studying the associations between TLR responsiveness patterns and clinical characteristics.

## Methods

### Study Population

For this study, 125 consecutive patients were enrolled who were scheduled for coronary angiography in the University Medical Hospital Utrecht between July 2009 and May 2011. As a control group, male individuals above 50 years without manifest coronary artery disease were included from the Military Hospital of the University Medical Hospital Utrecht. Exclusion criteria were currently present active inflammatory conditions, autoimmune disease, malignancies, use of immunosuppressive drugs and known hematological disorders. Clinical parameters were collected from case record forms including coronary angiograms. The ethics committee of the University Medical Centre Utrecht approved the protocol and all participants provided written informed consent prior to participation.

After 9 months, patients were approached to evaluate the occurrence of secondary events, after which endpoints were further verified. Primary endpoints were defined as the occurrence of cardiovascular death, myocardial infarction (MI), percutaneous coronary intervention (PCI), coronary artery bypass grafting (CABG) or cerebrovascular accident (CVA).

### Blood Sampling

Whole blood samples were collected in lithium-heparinized (LH) anti-coagulated tubes. To minimize the effects of the procedure on sample activation, patient samples were obtained prior to catheterization and administration of heparin therapy.

### Expression of Activation Markers CD11b and L-selectin after TLR Stimulation Measured by Flow Cytometry

Whole blood samples were stimulated directly after collection with increasing concentrations of either lipopolysaccharide (LPS; TLR4) (0.01, 0.1, 1, 10 ng/ml) or P3C (TLR2) (0.5, 5, 50, 500 ng/ml) for 15 min at 37°C and 5% CO_2_. PBS incubation served as a control to determine baseline expression. Stimulated samples were stained for CD14 (PE-Cy5, Beckman Coulter), CD11b (PE-Cy7, eBioscience) and L-selectin (ECD, Beckman Coulter) for 20 min. Expression levels of CD11b and L-selectin were determined within the CD14+ population by flow cytometry (FC500, Beckman Coulter). Average fluorescent intensity per cell is expressed as mean fluorescence intensity (MFI). A cut-off of at least 300 CD14+ monocytes was chosen to be included in the analysis.

### Dose-response Curves

Combined dose-response curves of patients and controls were created based on the mean CD11b and L-selectin expression per concentration to assess the effect of the presence of cardiovascular disease on TLR responsiveness (sigmoidal dose response curves with variable slope, Graphpad prism 5).

Baseline expression (after PBS stimulation) of CD11b and L-selectin varied significantly among individuals. To enable discrimination of response types (i.e. hypo−/hyperresponse) in relation to clinical characteristics, irrespective of baseline marker expression, delta values (difference in expression levels between baseline and after stimulation with each concentration of the ligand) were calculated to determine the actual TLR response. Subsequently, dose-response curves were created for each individual (sigmoidal dose response curves with variable slope, Graphpad prism 5). Dose response curves created from these values will be referred to as delta CD11b or delta L-selectin.

**Figure 1:**
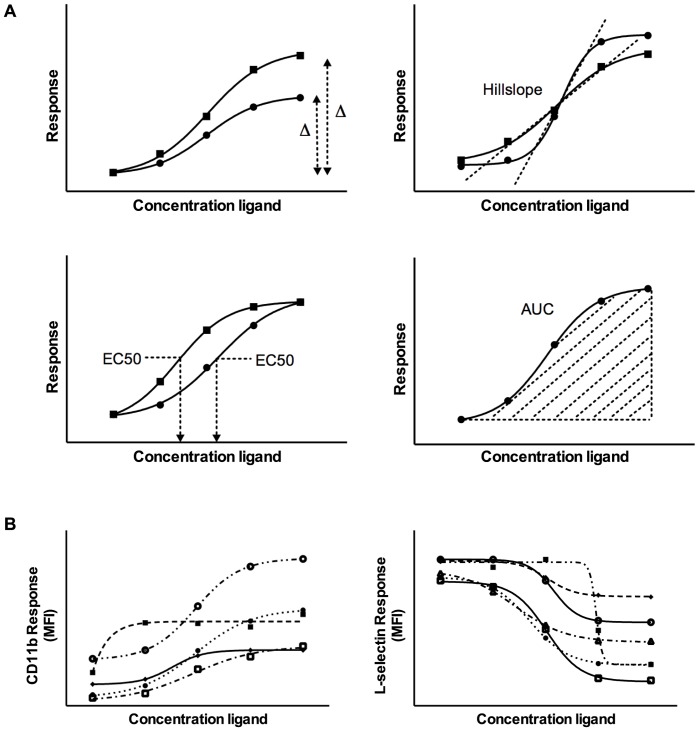
Derivatives of dose-response curves. A. From each dose-response curve, four different derivatives can be calculated to assess TLR-responsiveness: hillslope (maximum steepness of the curve), EC50 (ligand concentration resulting in half-maximum activation), area under the curve (AUC) and the delta (difference between minimum and maximum expression levels). B. Representative selection of CD11b and L-selectin response curves from individual patients showing high inter-individual variability after LPS stimulation.

The following derivatives were calculated for each curve (Figure 1A):

  - Hillslope = maximum steepness of the dose-response curve
  - EC50 = concentration of the ligand resulting in half-maximum activation
  - Area Under the Curve (AUC) = area under the dose-response curves
  - Delta = difference between baseline and maximum expression levels

Dose-response curves that were ambiguous or did not reach a plateau at high LPS concentrations resulting in an unreliable delta, AUC and EC50 were excluded (n = 18 for CD11b; n = 6 for L-selectin, all CAD patients). For the P3C stimulations we did not assess the different patterns in the dose response curves.

### Statistics

SPSS version 20.0 (Chicago, IL, USA) was used for all statistical analyses. Baseline characteristics of CAD patients and controls were compared with Chi-square, Fisher exact or Mann Whitney U test where appropriate. As responsiveness data were not normally distributed, non-parametric testing was performed. Dichotomous clinical variables were categorized in two groups and Mann-Whitney U test performed. For continuous clinical variables Spearman correlation was used. To determine the effect of risk factors and medication use on the measures of responsiveness, we executed a linear regression (enter model) comparing controls and CAD patients. In addition, a backward linear regression was performed to assess which key patient phenotypes (risk factors and clinical determinants) drive the differences in TLR responsiveness in CAD patients. A two-sided p-value <0.05 was considered statistically significant.

## Results

Baseline clinical characteristics of the 125 coronary artery disease (CAD) patients and 28 controls are reported in Table 1. Despite all attempts to include age-matched subjects, controls were significantly younger (average age: 53.3 vs. 61.3 years). Furthermore, controls presented with significantly less risk factors and less medication use was reported.

**Table 1:** Baseline characteristics of CAD patients and controls.

	Patient cohort	Controls	p-value
	(n = 125)	(n = 28)	
**Risk factors**			
Age, mean ± SD, y	61.3±9.3	53.3±2.8	<0.001
Male gender	98 (78.4%)	28 (100%)	0.008
Current smoker	16 (12.8%)	6 (21.4%)	0.241
Diabetes	16 (12.8%)	0 (0%)	0.077
Hypertension	83 (66.4%)	9 (32.1%)	0.013
Dyslipidemia	78 (62.4%)	5 (17.9%)	<0.001
BMI, mean ± SD, kg/m^2^	27.9±5.2	27.2±3.1	0.733
eGFR (MDRD), mean ± SD, ml/min/1.73 m^2^	81.5±32.7	119.3±23.1	0.016
Previous PCI/MI	60 (48.0%)	**−**	**−**
**Clinical presentation**			
Confirmed diagnosis			
SA	113 (90.4%)	**−**	**−**
UA/NSTEMI	12 (9.6%)	**−**	**−**
**Angiographic parameters**			
Multi vessel disease (stenosis >50%)	70 (56.0%)	**−**	**−**
Highest degree of stenosis >90%	49 (39.2%)	**−**	**−**
Occlusion	33 (26.4%)	**−**	**−**
**Follow-up**			
Primary endpoint	17 (13.6%)	**−**	**−**
**Medication use**			
Beta-blocker	103 (82.4%)	4 (14.3%)	<0.001
ACE inhibitor	55 (44.0%)	3 (10.7%)	0.005
Ca-antagonist	34 (27.2%)	0 (0%)	0.004
ATII antagonist	21 (16.8%)	2 (7.1%)	0.531
Statin	97 (77.6%)	1 (3.6%)	<0.001
ASA	113 (90.4%)	1 (3.6%)	<0.001
Clopidogrel	106 (84.8%)	0 (0%)	<0.001

BMI, body mass index; eGFR, estimated glomerular filtration rate; PCI, percutaneous coronary intervention; MI, myocardial infarction; SA, stable angina pectoris; UA, unstable angina pectoris; NSTEMI, non-ST-elevated myocardial infarction; ASA, acetylsalicylic acid.

Dose-response curves of the activation markers CD11b and L-selectin were constructed for each individual after stimulation with increasing concentrations of LPS and P3C. As demonstrated in Figure 1B, dose-response curves markedly varied among individuals.

### TLR Response CAD Patients vs. Controls

**Figure 2:**
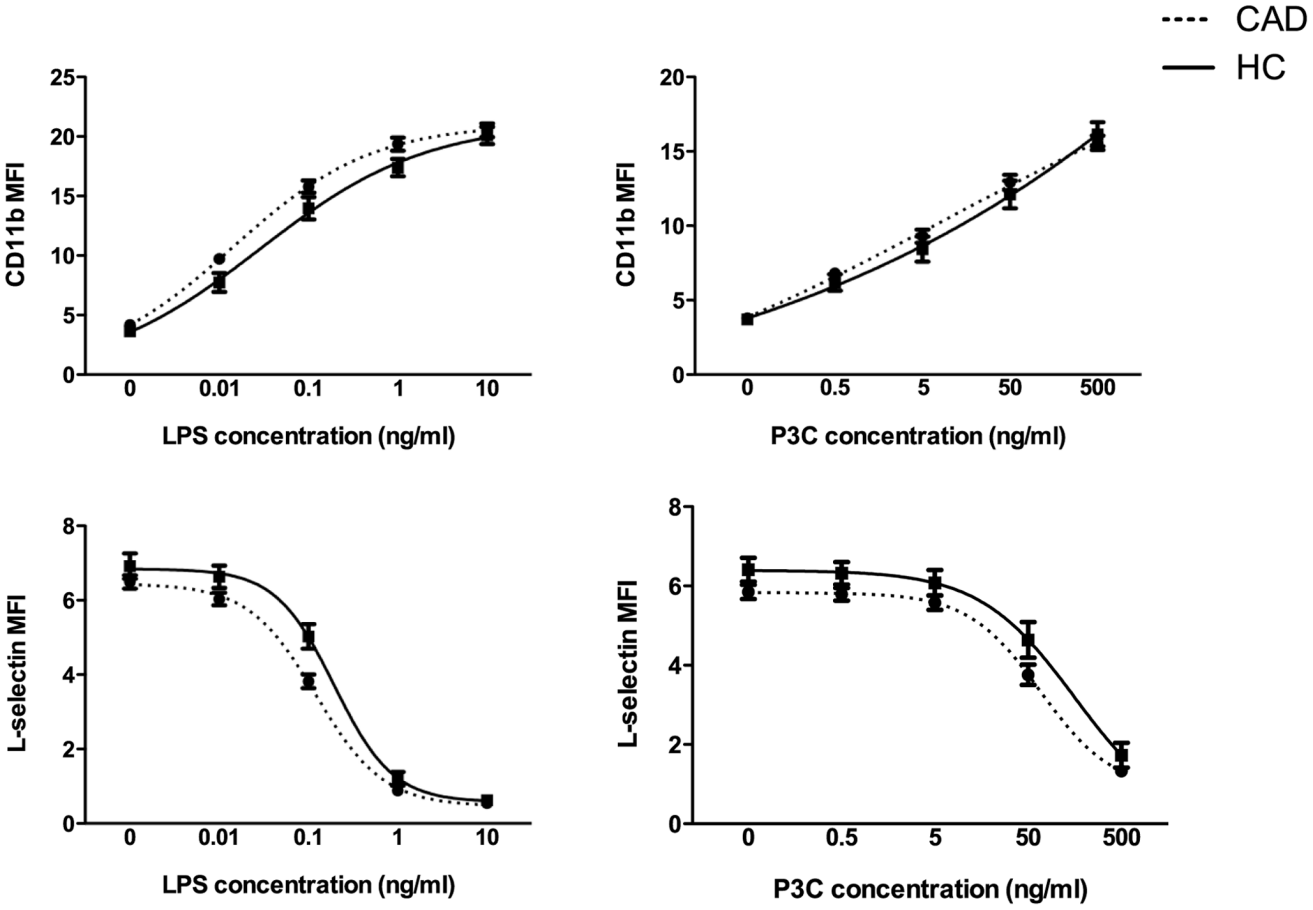
Dose-response curves of CAD patients and controls. Mean dose-response curves of CAD patients (dashed line) vs. controls (continuous line) for CD11b (upper panel) and L-selectin (lower panel). Samples were either stimulated with increasing concentrations of LPS (left hand panel) or P3C (right hand panel). PBS stimulation served as a control (LPS concentration = 0). Concentrations are log-transformed. Data are presented as mean ± S.E.M.

In all subjects, CD11b expression increased and L-selectin expression decreased after TLR stimulation. Combined dose-response curves of controls and CAD patients are illustrated in Figure 2. CAD patients showed to be more responsive to TLR ligands, indicated by a higher CD11b expression particularly at intermediate concentrations and a faster and more extensive L-selectin shedding.

Single dose stimulation only significantly differed between controls and CAD patients for L-selectin after LPS 0.1 ng/ml stimulation (Table 2). Dose-response curves showed that CAD patients have a significantly lower hillslope for L-selectin and a lower EC50 for both markers compared to controls. This indicates that circulating monocytes of patients need a lower trigger to become activated, but once activated, the response of controls is more extensive (Table 2). The differences in LPS-induced L-selectin response between controls and CAD patients (both after single dose stimulation and derivatives of dose-response curves) become even more evident after correction for risk factors and medication use (Table 3). Next to the differences between CAD patients and controls, we also investigated whether the observed variation in TLR-responsiveness among patients could be explained by clinical characteristics, such as cardiovascular risk factors, atherosclerotic disease severity and secondary cardiovascular events, by analyzing single dose stimulations and dose response curves.

**Table 2:** CD11b and L-selectin expression of CAD patients and controls.

CD11b	Controls	CAD patients	p-value	L-selectin	Controls	CAD patients	p-value
PBS	3.4 [2.0]	3.0 [1.8]	0.126	PBS	6.3 [2.0]	5.8 [2.9]	0.095
LPS 0.01	6.1 [7.2]	9.3 [7.1]	0.118	LPS 0.01	6.6 [1.9]	6.1 [2.9]	0.148
LPS 0.1	14.3 [5.2]	15.4 [7.8]	0.178	LPS 0.1	5.1 [2.8]	3.7 [2.9]	0.003*
LPS 1	17.6 [5.1]	17.9 [6.9]	0.592	LPS 1	0.82 [1.1]	0.66 [0.5]	0.153
LPS 10	19.0 [5.6]	19.1 [8.1]	0.694	LPS 10	0.45 [0.3]	0.47 [0.2]	0.438
P3C 0.5	5.4 [3.1]	6.0 [4.2]	0.688	P3C 0.5	6.2 [1.9]	5.6 [2.4]	0.161
P3C 5	7.5 [4.8]	8.4 [8.3]	0.434	P3C 5	6.2 [1.9]	5.7 [2.7]	0.177
P3C 50	11.7 [5.9]	12.3 [9.0]	0.540	P3C 50	5.0 [4.3]	3.7 [5.5]	0.092
P3C 500	16.6 [4.1]	15.2 [5.9]	0.243	P3C 500	0.96 [1.9]	0.64 [1.2]	0.120
LPS Hillslope	1.01 [0.67]	0.78 [0.46]	0.162	LPS Hillslope	1.86 [9.3]	1.39 [1.01]	0.037*
LPS EC50	0.06 [0.07]	0.03 [0.05]	0.003**	LPS EC50	0.21 [0.73]	0.11 [0.14]	0.004**
LPS AUC	34.4 [13.6]	36.9 [15.8]	0.469	LPS AUC	10.2 [7.4]	11.4 [5.3]	0.424
LPS Delta	15.6 [5.0]	16.0 [7.0]	0.689	LPS Delta	6.1 [2.5]	5.9 [2.6]	0.416

Expression levels of CD11b and L-selectin of CAD patients vs. controls at baseline and after TLR stimulation. Data are presented as median [IQR].

* p<0.05.

** p<0.01.

**Table 3:** CD11b and L-selectin expression of CAD patients and controls adjusted for risk factors and medication use.

CD11b	B	β	p-value	L-selectin	B	β	p-value
PBS	**−**0.653	**−**0.112	0.467	PBS	**−**1.709	**−**0.344	0.030*
LPS 0.01	3,267	0.260	0.089	LPS 0.01	**−**1.696	**−**0.338	0.033*
LPS 0.1	2.077	0.152	0.307	LPS 0.1	**−**2.756	**−**0.520	0.001**
LPS 1	1.994	0.143	0.344	LPS 1	**−**1.350	**−**0.765	<0.001**
LPS 10	2.650	0.184	0.220	LPS 10	**−**0.648	**−**0.971	<0.001**
P3C 0.5	**−**0.024	**−**0.002	0.987	P3C 0.5	**−**1.338	**−**0.295	0.064
P3C 5	**−**0.448	**−**0.036	0.814	P3C 5	**−**1.928	**−**0.372	0.014*
P3C 50	0.095	0.007	0.966	P3C 50	**−**1.386	**−**0.194	0.218
P3C 500	1.566	0.118	0.441	P3C 500	**−**0.815	0.196	0.228
LPSHillslope	**−**1.735	**−**0.279	0.101	LPSHillslope	**−**3.452	**−**0.445	0.005**
LPS EC50	0.291	0.167	0.324	LPS EC50	**−**0.421	**−**0.624	<0.001**
LPS AUC	8.798	0.293	0.080	LPS AUC	0.753	**−**0.063	0.702
LPS Delta	2.803	0.229	0.169	LPS Delta	**−**0.902	**−**0.182	0.271

Differences in CD11b and L**−**selectin expression levels between CAD patients and controls after correction for risk factors and medication use further defined as age, gender, smoking, diabetes, hypertension, dyslipidemia, BMI, eGFR, betablocker, ACE inhibitors, Ca-antagonist, ATII antagonist, statin, ASA, clopidogrel. Data are presented as median [IQR].

* p<0.05.

** p<0.01.

### Single dose TLR Stimulation in CAD Patients

**Figure 3:**
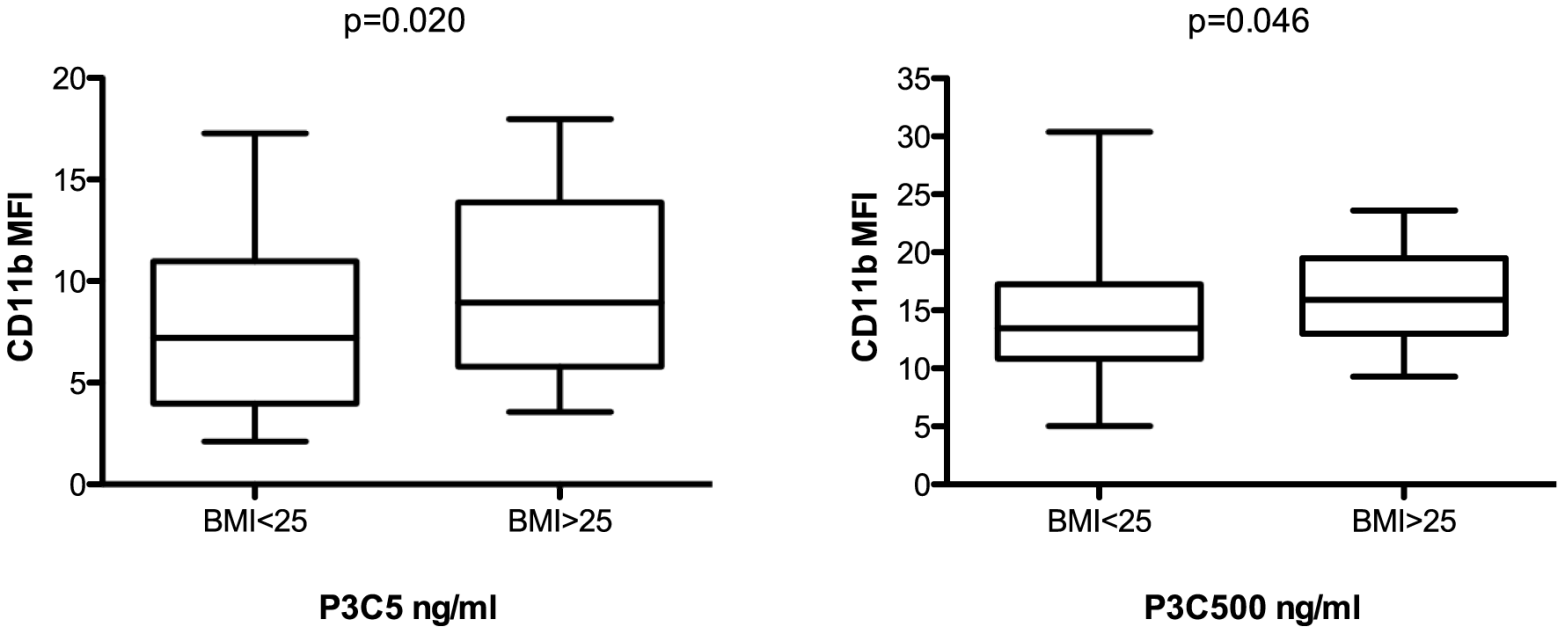
Single dose TLR response in relation to BMI. CD11b expression after stimulation with P3C 5 ng/ml (left) and P3C 500 ng/ml (right) was significantly higher in patients with a BMI>25 as compared to normal weight patients (BMI<25). Whiskers are presented as 5–95 percentile. Data were statistically tested with a Mann Whitney U test.

Two fixed concentrations of LPS (0.01 and 10 ng/ml), P3C (5 and 500 ng/ml) and PBS control were selected to study differences between patients in TLR-induced CD11b (Table S1A) and L-selectin (Table S1B) expression. Patients with a Body Mass Index (BMI) above 25 showed a significantly higher CD11b expression after TLR2 stimulation than normal-weight subjects (P3C 5: MFI 9.0 vs. MFI 7.2, p = 0.020; P3C 500: MFI 15.9 vs. MFI 13.5, p = 0.046) (Figure 3). Also when BMI was tested as a continuous variable, a strong correlation could be observed with TLR2-induced CD11b expression (data not shown). Other continuous parameters, such as age, CRP, and lipids, did not show relevant correlations with TLR-induced CD11b and L-selectin expression (data not shown).

To determine which clinical characteristics and risk factors act as key factors in the TLR response we performed a backward regression model. The higher CD11b expression after TLR2 stimulation in patients with a BMI>25 remained significant after correction for other factors. Additionally, we observed higher CD11b expression levels in unstable CAD patients as compared to patients with stable angina after correction for risk factors and angiographic parameters. These differences reached significance for TLR2 stimulations.

### Dose-response Curves in CAD Patients

**Figure 4:**
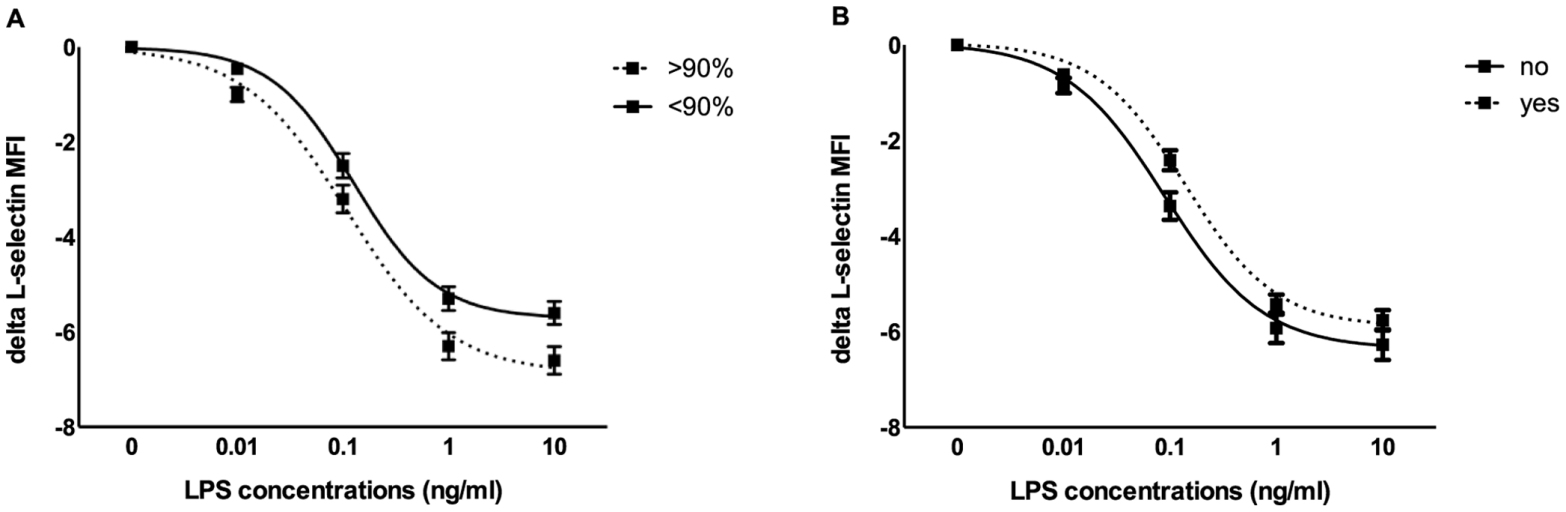
Dose-response curves of CAD patients in relation to clinical characteristics. A. L-selectin dose-response curves of patients with severe (>90%) coronary stenosis showed a significantly reduced hillslope and an increased AUC and delta as compared to patients with <90% stenosis. B. Responsiveness of L-selectin in patients with arterial hypertension was significantly less compared to normotensive patients indicated by a reduced AUC and a higher EC50.

Within the CAD patient cohort, CD11b response after LPS stimulation showed a significantly higher hillslope in females (Table S2A). For L-selectin, significant differences were observed for patients with hypertension, dyslipidemia, occlusion and degree of stenosis (Table S2B). Patients with a stenosis >90% had a lower median hillslope (1.34 vs. 1.67, p = 0.007) and a higher median AUC (12.4 vs. 10.5, p = 0.018) and delta (6.6 vs. 5.6, p = 0.016) (Figure 4A). Furthermore, patients with hypertension showed a lower responsiveness as indicated by a higher EC50 (0.09 vs. 0.14, p = 0.037) and lower AUC (12.3 vs. 10.9, p = 0.042) (Figure 4B). Continuous clinical parameters did not show any significant correlations with dose-responsiveness (data not shown). After correction for risk factors and angiographic parameters, we could no longer detect consistent associations between specific clinical characteristics and the different readouts for TLR response.

## Discussion

Stimulation of TLRs results in the production and release of pro-inflammatory cytokines and promotes adhesion of circulating leukocytes, which are essential in the development and progression of atherosclerotic plaques. In the present study, we measured TLR-induced CD11b and L-selectin expression on circulating monocytes to assess whether changes of these fast responding markers are able to distinguish between CAD patients and controls or among patients. Furthermore, we hypothesized that detailed TLR-induced dose-responsiveness patterns could be a better measure of activation response than single dose TLR stimulation.

We show that the up-regulation of CD11b expression and shedding process of L-selectin already occurs within 15 minutes after both TLR2 and TLR4 stimulation in all individuals. In addition, our data show that dose-response curves, but not single dose TLR stimulation, reveal significant differences between controls and CAD patients after univariate testing. Circulating monocytes of CAD patients seemed to be more easily activated as reflected by the significantly lower EC50 for both CD11b and L-selectin. In contrast, baseline and maximum expression levels did not differ between both groups, supporting an added value of dose response curves rather than single dose stimulation for discrimination of CAD patients and controls. However, multivariate regression analysis showed that also single dose LPS stimulations as measured by L-selectin expression, as well as the hillslope and EC50 calculated from the dose-response curves significantly differ between CAD patients and controls. This suggests that the differences in L-selectin response after LPS stimulation were masked by risk profiles and medication use. Thus, LPS induced L-selectin shedding is capable of distinguishing between controls and CAD patients, either after single dose response or dose response curve derivatives.

CD11b and L-selectin response after TLR stimulation varied widely among patients. Hence, we speculated that this wide variation might provide information about the individual disease-state or the likelihood of future events. One low and one high concentration of LPS (0.1 and 10 ng/ml) and P3C (5 and 500 ng/ml) were selected to explore single dose CD11b and L-selectin responsiveness among CAD patients. Median TLR2-induced CD11b expression was higher in patients with a BMI above 25 compared to normal-weight subjects for single dose TLR stimulation for both concentrations of P3C. This concurs with our previous study showing an increased TLR-induced TNFα response in obese patients undergoing PCI or carotid endarterectomy (CEA) and an increased CD11b response after TLR stimulation in patients undergoing CEA [18]. Other clinical and angiographic parameters did not show any significant differences after univariate testing, which is in line with Servi et al., who also found no association of baseline monocyte CD11b expression with clinical factors. However, in their study, CD11b expression tended to be higher in patients with angiographic complex lesion morphology [19]. Multivariate testing showed that unstable angina or NSTEMI patients had significantly higher CD11b levels than stable angina patients. This suggests that CD11b may not be a good marker to discriminate subgroups of stable angina patients, but might reflect more advanced atherosclerosis.

Data about the role of membrane bound L-selectin in cardiovascular patients is scarce. Several studies suggest an important role of L-selectin in atherogenesis, but data from clinical studies are limited [1], [20], [21]. In patients with ischemic stroke, soluble L-selectin levels were significantly increased suggesting a higher circulating cell response accompanied by L-selectin shedding [20].

Derivatives of L-selectin dose-response curves show significant differences for hypertension, dyslipidemia, occlusion and degree of stenosis, the association with the latter being the strongest. Patients with a coronary stenosis above 90% had a significantly lower hillslope and a higher AUC and delta, suggesting a higher responsiveness in these patients. This observation is in line with our previous study where we describe a higher TLR-induced TNFα response in patients with a stenosis above 90% [8]. Surprisingly, patients with hypertension displayed a lower TLR response. However, antihypertensive drugs used by the majority of these patients could explain this effect. Furthermore, our data show that more severely diseased patients based on coronary angiograms and clinical presentation, have a lower hillslope for both activation markers and a higher AUC and delta for L-selectin. After multivariate testing, none of the univariate associations remained valid except for the lower AUC of hypertensive patients.

The significant but subtle differences in TLR-induced CD11b and L-selectin responsiveness will likely not suffice to discriminate between individual patients at low or high risk to suffer from CAD in a clinical setting. The majority of patients in our cohort presented with stable angina, and therefore the differences between these individuals might be too small for these markers to clearly discriminate between individual patients in both groups. This is supported by previous studies in which baseline CD11b expression was up-regulated in patients with UA and ACS but no significant differences were found between SA and controls [16], [22].

In previous studies, others and our group have demonstrated that processes occurring in the vascular wall can affect the TLR-responsiveness of circulating cells as measured by cytokine release. In these studies, a reduction in TLR-induced cytokine release shortly after PCI, vascular surgery and myocardial ischemia was observed [8], [23], [24], suggesting that vascular injury and ischemia influences the TLR-response of circulating cells. Furthermore, it has been reported that TLR-response alters with progression of atherosclerotic disease [8] and in patients with unstable angina as compared to stable angina [6], [7]. We show that CD11b and L-selectin expression levels after TLR stimulation are also capable of discriminating between patients, even though they are potentially less sensitive than TLR-induced cytokine levels.

In conclusion, dose-response curves and single dose stimulations of LPS-induced L-selectin expression can discriminate between controls and CAD patients. Furthermore, single dose stimulations and dose-response curves of CD11b and L-selectin expression provide diverse information. Where more associations with clinical characteristics were found for CD11b after single dose stimulation, differences in L-selectin expression were more evident for dose-response curves. Based on this study we cannot conclude yet which one would be the most informative.

### Limitations

For this study, multiple concentrations of TLR ligands were used to sort out dose dependent effects of TLR ligands in association with clinical characteristics. Our study showed positive associations for BMI and TLR2-induced CD11b expression, clinical presentation and CD11b expression and stenosis degree and L-selectin dose response derivatives, but correction for multiple testing probably would have limited the effect size. This study should therefore be considered as an exploratory exercise to examine clinical determinants that associate with TLR responsiveness. Our inferences are weakened by the large number of comparisons and lack of significance when corrected for multiple testing and therefore require validation in a study with a more targeted approach.

To improve precision and accuracy of the outcome, this study needs to be validated preferably with additional dosages in the intermediate range. This would result in more reliable dose-response curves and derivatives calculated from this. Dose-response curves after TLR2 stimulation were excluded because of too many unreliable curves. In future studies, higher concentrations of P3C, next to additional dosages in the intermediate range, will be needed to investigate TLR2 induced dose-response curves of CD11b and L-selectin in atherosclerotic patients.

## Supporting Information

Table S1 **Single dose TLR response and clinical characteristics.** CD11b (A) and L-selectin (B) expression after single dose TLR stimulation in relation to clinical baseline characteristics. For the multivariate analyses a backward linear regression model including age, gender, smoking, diabetes, hypertension, dyslipidemia, BMI, eGFR, previous coronary event, clinical presentation, number of diseased vessels, degree of stenosis, occlusion was used. Data are presented as median [IQR]. ∧p<0.05 in univariate analysis, *p<0.05 in multivariate analysis.
(DOCX)

Table S2 **Dose-response curves and clinical characteristics.** Dose-response curves of CD11b (A) and L-selectin (B) in relation to baseline clinical characteristics. For the multivariate analyses a backward linear regression model including age, gender, smoking, diabetes, hypertension, dyslipidemia, BMI, eGFR, previous coronary event, clinical presentation, number of diseased vessels, degree of stenosis, occlusion was used. Data are presented as median [IQR]. ∧p<0.05 in univariate analysis, *p<0.05 in multivariate analysis.
(DOCX)

## References

[1] Ross R (1999) Atherosclerosis - an inflammatory disease. N Engl J Med 340: 115–126. 9887164 10.1056/NEJM199901143400207

[2] Edfeldt K, Swedenborg J, Hansson GK, Yan Z (2002) Expression of Toll-like receptors in human atherosclerotic lesions: a possible pathway for plaque activation. Circulation 105: 1158–1161. 11889007

[3] Vink A, Schoneveld AH, Meer JJ van der, Middelaar BJ van, Sluijter JPG, et al (2002) In vivo evidence for a role of Toll-like receptor 4 in the development of intimal lesions. Circulation 106: 1985–1990. 12370224 10.1161/01.cir.0000032146.75113.ee

[4] Ishikawa Y, Satoh M, Itoh T, Minami Y, Takahashi Y, et al (2008) Local expression of Toll-like receptor 4 at the site of ruptured plaques in patients with acute myocardial infarction. Clinical Sci (Lond) 115: 133–140. 18282141 10.1042/CS20070379 PMC2552974

[5] Ashida K, Miyazaki K, Takayama E, Tsujimoto H, Ayaori M, et al (2005) Characterization of the expression of TLR2 (Toll-like receptor 2) and TLR4 on circulating monocytes in coronary artery disease. J Atheroscler Thromb 12: 53–60. 15725697 10.5551/jat.12.53

[6] Methe H, Kim J-O, Kofler S, Weis M, Nabauer M, et al (2005) Expansion of circulating Toll-like receptor 4-positive monocytes in patients with acute coronary syndrome. Circulation 111: 2654–2661. 15883205 10.1161/CIRCULATIONAHA.104.498865

[7] Liuzzo G, Angiolillo DJ, Buffon A, Rizzello V, Colizzi C, et al (2001) Enhanced response of blood monocytes to in vitro lipopolysaccharide-challenge in patients with recurrent unstable angina. Circulation 103: 2236–2241. 11342470 10.1161/01.cir.103.18.2236

[8] Versteeg D, Hoefer IE, Schoneveld AH, De Kleijn DP V, Busser E, et al (2008) Monocyte Toll-like receptor 2 and 4 responses and expression following percutaneous coronary intervention: association with lesion stenosis and fractional flow reserve. Heart 94: 770–776. 17686802 10.1136/hrt.2007.117259

[9] Arnaout M (1990) Structure and function of the leukocyte adhesion molecules CD11/CD18. Blood 75: 1037–1050. 1968349

[10] Mazzone A, Ricevuti G (1995) Leukocyte CD11/CD18 integrins: biological and clinical relevance. Haematologica 80: 161–175. 7628754

[11] Rainer TH (2002) L-selectin in health and disease. Resuscitation 52: 127–141. 11841880 10.1016/s0300-9572(01)00444-0

[12] Wedepohl S, Beceren-Braun F, Riese S, Buscher K, Enders S, et al (2011) L-Selectin - a dynamic regulator of leukocyte migration. Eur J Cell Biol 91: 257–264. 21546114 10.1016/j.ejcb.2011.02.007

[13] Zhou X, Gao X-P, Fan J, Liu Q, Anwar KN, et al (2005) LPS activation of Toll-like receptor 4 signals CD11b/CD18 expression in neutrophils. Am J Physiol Lung Cell Mol Physiol 288: L655–62. 15563689 10.1152/ajplung.00327.2004

[14] Gomes NE, Brunialti MKC, Mendes ME, Freudenberg M, Galanos C, et al (2010) Lipopolysaccharide-induced expression of cell surface receptors and cell activation of neutrophils and monocytes in whole human blood. Braz J Med Biol Res 43: 853–858. 20721471 10.1590/s0100-879x2010007500078

[15] Kassirer M, Zeltser D, Prochorov V, Schoenman G, Frimerman A, et al (1999) Increased expression of the CD11b/CD18 antigen on the surface of peripheral white blood cells in patients with ischemic heart disease: further evidence for smoldering inflammation in patients with atherosclerosis. Am Heart J 138: 555–559. 10467208 10.1016/s0002-8703(99)70160-2

[16] Choi Y, Lee W, Lee Y, Kim JK, Lee SY, et al (2000) Correlation between monocyte and T-lymphocyte activation markers in patients. Jpn Heart J 41: 605–615. 11132167 10.1536/jhj.41.605

[17] Mazzone A, Servi S De, Mazzucchelli I, Fossati G, Gritti D, et al (1997) Increased expression of CD11b/CD18 on phagocytes in ischaemic disease: a bridge between inflammation and coagulation. Eur J Clin Invest 27: 648–652. 9279527 10.1046/j.1365-2362.1997.1610710.x

[18] Scholtes VPW, Versteeg D, De Vries JPPM, Hoefer IE, Schoneveld AH, et al (2011) Toll-like receptor 2 and 4 stimulation elicits an enhanced inflammatory response in human obese patients with atherosclerosis. Clinical Sci (Lond) 121: 205–214. 21446916 10.1042/CS20100601

[19] De Servi S, Mazzone A, Ricevuti G, Mazzucchelli I, Fossati G, et al (1995) Clinical and angiographic correlates of leukocyte activation in unstable angina. J Am Coll Cardiol 26: 1146–1150. 7594025 10.1016/0735-1097(95)00308-8

[20] Wei Y-S, Lan Y, Meng L-Q, Nong L-G (2011) The association of L-selectin polymorphisms with L-selectin serum levels and risk of ischemic stroke. J Thromb and thrombolysis 32: 110–115. 21465128 10.1007/s11239-011-0587-4

[21] Eriksson EE, Xie X, Werr J, Thoren P, Lindbom L (2001) Importance of primary capture and L-selectin-dependent secondary capture in leukocyte accumulation in inflammation and atherosclerosis in vivo. J Exp Med 194: 205–218. 11457895 10.1084/jem.194.2.205 PMC2193449

[22] Mazzone A, De Servi S, Ricevuti G, Mazzucchelli I, Fossati G, et al (1993) Increased expression of neutrophil and monocyte adhesion molecules in unstable coronary artery disease. Circulation 88: 358–363. 8101771 10.1161/01.cir.88.2.358

[23] Elsenberg EH a M, Versteeg D, Sels J-W, Vlaar P-JJ, Hobbelink MGG, et al (2012) Inducible cardiac ischaemia is related to a decrease in the whole-blood Toll-like receptor 2 and 4 response. Clinical Sci (Lond) 122: 527–533. 22188581 10.1042/CS20110323

[24] Versteeg D, Dol E, Hoefer IE, Flier S, Buhre WF, et al (2009) Toll-like receptor 2 and 4 response and expression on monocytes decrease rapidly in patients undergoing arterial surgery and are related to preoperative smoking. Shock 31: 21–27. 18650783 10.1097/SHK.0b013e31817d43bf

