# Supplementary material for: Toll-Like Receptor Induced CD11b and L-Selectin Response in Patients with Coronary Artery Disease
Source: PLoS One. 2013 Apr 3;8(4):e60467. doi: 10.1371/journal.pone.0060467 (PMC3616095; doi:10.1371/journal.pone.0060467)
Supplement: Table S1 — Single dose TLR response and clinical characteristics. CD11b (A) and L-selectin (B) expression after single dose TLR stimulation in relation to clinical baseline characteristics. For the multivariate analyses a backward linear regression model including age, gender, smoking, diabetes, hypertension, dyslipidemia, BMI, eGFR, previous coronary event, clinical presentation, number of diseased vessels, degree of stenosis, occlusion was used. Data are presented as median [IQR]. ∧p<0.05 in univariate analysis, *p<0.05 in multivariate analysis. (DOCX) [file pone.0060467.s001.docx]

**Table S1A. CD11b expression**

|  | **PBS** | **LPS 0.01 ng/ml** | **LPS 10 ng ml** | **P3C 5 ng/ml** | **P3C 500 ng ml** |
| --- | --- | --- | --- | --- | --- |
|  | **N=124** | **N=122** | **N=122** | **N=124** | **N=123** |
| **Risk Factors** |  |  |  |  |  |
| Age (<60/>60) | 3.1 [1.9] vs. 2.9 [1.6] | 10.2 [6.9] vs. 9.0 [7.5] | 20.3 [8.6] vs. 18.9 [7.1] | 9.0 [7.7] vs. 7.5 [8.4] | 15.4 [6.1] vs. 15.0 [6.4] |
| Gender (m/f) | 2.9 [1.8] vs. 3.0 [1.6] | 9.4 [6.8] vs. 9.2 [9.1] | 19.3 [7.8] vs. 18.4 [7.8] | 8.5 [8.3] vs. 7.8 [7.8] | 15.0 [5.4] vs. 15.9 [9.0] |
| Current smoker (n/y) | 2.9 [1.8] vs. 3.6 [2.1] | 9.1 [7.8] vs. 9.8 [5.5] | 19.3 [8.2] vs. 17.8 [8.2] | 8.4 [8.5] vs. 7.3 [6.9] | 15.2 [6.9] vs. 12.9 [6.4] |
| Diabetes (n/y) | 2.9 [1.8] vs. 3.6 [1.9]* | 9.3 [7.2] vs. 9.3 [7.1] | 19.3 [8.4] vs. 18.9 [5.4] | 8.2 [7.9] vs. 10.9 [9.5] | 15.3 [5.9] vs. 14.7 [7.7] |
| Hypertension (n/y) | 2.8 [1.7] vs. 3.0 [2.2] | 8.8 [4.8] vs. 9.6 [8.6] | 18.9 [7.4] vs. 19.7 [8.4] | 8.2 [6.5] vs. 8.5 [8.8] | 14.1 [4.8] vs. 16.0 [6.8] |
| Dyslipidemia (n/y) | 3.0 [2.1] vs. 3.0 [1.7] | 10.6 [8.8] vs. 9.0 [6.7] | 19.4 [8.4] vs. 18.8 [7.5] | 9.0 [6.9] vs. 8.2 [8.8] | 15.4 [4.7] vs. 15.2 [6.8] |
| BMI (<25/>25) | 2.8 [1.6] vs. 3.0 [1.8] | 9.1 [6.6] vs. 9.6 [7.1]* | 18.8 [7.6] vs. 19.7 [7.5] | 7.2 [7.0] vs. 9.0 [8.1]^ | 13.5 [6.4] vs. 15.9 [6.5]^* |
| eGFR (<60/>60) | 2.7 [1.1] vs. 3.0 [1.9] | 9.1 [4.8] vs. 9.7 [7.5] | 18.4 [7.4] vs. 19.3 [7.9] | 9.0 [9.8] vs. 8.4 [8.1] | 14.7 [5.5] vs. 15.4 [7.1] |
| Previous coronary event (n/y) | 3.0 [1.8] vs. 3.0 [1.7] | 9.5 [7.1] vs. 9.1 [7.4] | 18.8 [8.8] vs. 19.8 [7.0] | 7.9 [7.0] vs. 8.7 [8.8] | 14.9 [6.3] vs. 15.6 [6.8] |
|  |  |  |  |  |  |
| **Clinical presentation** |  |  |  |  |  |
| (SA/UA+NSTEMI) | 3.0 [1.7] vs. 3.0 [6.2]* | 9.1 [7.4] vs. 11.1 [4.9] | 19.0 [8.1] vs. 21.9 [6.5] | 7.9 [7.7] vs. 13.4 [8.6]* | 15.0 [5.9] vs. 16.7 [6.8]* |
|  |  |  |  |  |  |
| **Angiographic parameters** |  |  |  |  |  |
| No of vessels (single/multi) | 2.9 [2.1] vs. 2.9 [1.5] | 9.1 [7.7] vs. 9.6 [6.7] | 19.0 [9.3] vs. 19.0 [7.5] | 8.5 [9.1] vs. 8.3 [9.6] | 13.9 [7.6] vs. 15.4 [5.5] |
| Degree stenosis (<90%/>90%) | 3.0 [1.6] vs. 2.8 [1.7] | 9.1 [7.2] vs. 9.2 [6.9] | 19.0 [7.4] vs. 18.6 [8.3] | 8.2 [8.1] vs. 7.9 [8.8] | 15.0 [5.9] vs. 14.1 [6.1] |
| Occlusion (n/y) | 3.0 [1.6] vs. 2.7 [2.6] | 9.1 [7.5] vs. 10.0 [5.7] | 19.0 [8.0] vs. 19.3 [7.6] | 7.8 [7.9] vs. 10.2 [7.0] | 15.1 [6.6] vs. 16.0 [6.0] |
|  |  |  |  |  |  |
| **Event score** |  |  |  |  |  |
| (no/primary endpoint) | 2.9 [1.8] vs. 2.8 [2.5] | 10.1 [8.4] vs. 8.9 [2.8] | 19.0 [8.3] vs. 20.1 [6.9] | 8.5 [8.6] vs. 9.4 [8.5] | 15.2 [5.4] vs.16.3 [6.7] |

BMI, body mass index; eGFR, estimated glomerular filtration rate; SA, stable angina pectoris; UA, unstable angina pectoris; NSTEMI, non-ST-elevated myocardial infarction, Previous coronary event = previous PCI and previous MI combined.

|  | **PBS** | **LPS 0.01 ng/ml** | **LPS 10 ng ml** | **P3C 5 ng/ml** | **P3C 500 ng ml** |
| --- | --- | --- | --- | --- | --- |
|  | **N=125** | **N=123** | **N=123** | **N=125** | **N=125** |
| **Risk Factors** |  |  |  |  |  |
| Age (<60/>60) | 5.1 [2.5] vs. 6.2 [2.8]^* | 5.7 [3.0] vs. 6.3 [2.8] | 0.45 [0.14] vs. 0.53 [0.20]^ | 5.2 [2.7] vs. 6.0 [2.7] | 0.55 [1.0] vs. 0.76 [1.2] |
| Gender (m/f) | 5.8 [3.0] vs. 5.8 [2.8] | 6.1 [2.9] vs. 5.6 [2.7] | 0.47 [0.16] vs. 0.52 [0.19] | 5.5 [2.7] vs. 6.0 [2.2] | 0.67 [1.3] vs. 0.57 [0.7] |
| Current smoker (n/y) | 5.8 [3.2] vs. 5.2 [1.8] | 6.2 [3.1] vs. 5.1 [1.3] | 0.47 [0.16] vs. 0.51 [0.20] | 5.8 [3.2] vs. 5.3 [1.7] | 0.61 [1.1] vs. 1.05 [1.4] |
| Diabetes (n/y) | 5.8 [2.8] vs. 6.1 [3.2] | 6.1 [2.9] vs. 5.7 [2.5] | 0.47 [0.16] vs. 0.49 [0.23] | 5.8 [2.6] vs. 5.3 [3.9] | 0.61 [1.2] vs. 0.70 [1.2] |
| Hypertension (n/y) | 6.1 [3.4] vs. 5.8 [2.5] | 6.4 [3.1] vs. 5.7 [2.7] | 0.47 [0.15] vs. 0.48 [0.18] | 5.3 [2.8] vs. 5.8 [2.9] | 0.61 [1.7] vs. 0.70 [1.1] |
| Dyslipidemia (n/y) | 5.8 [2.8] vs. 5.8 [2.8] | 6.0 [3.0] vs. 6.1 [2.9] | 0.47 [0.18] vs. 0.48 [0.17] | 5.6 [3.1] vs. 5.7 [2.6] | 0.89 [1.5] vs. 0.61 [1.0] |
| BMI (<25/>25) | 5.8 [2.8] vs. 5.8 [3.0] | 6.2 [2.7] vs. 6.0 [3.0] | 0.55 [0.23] vs. 0.47 [0.15] | 5.9 [2.1] vs. 5.5 [3.1] | 0.79 [1.0] vs. 0.61 [1.3] |
| eGFR (<60/>60) | 6.7 [2.7] vs. 5.8 [2.7] | 6.5 [3.4] vs. 6.0 [2.8] | 0.47 [0.17] vs. 0.47 [0.16] | 6.8 [4.5] vs. 5.6 [2.6] | 0.49 [0.6] vs. 0.64 [1.2] |
| Previous coronary event (n/y) | 5.8 [3.6] vs. 6.0 [2.7] | 6.0 [2.6] vs. 6.1 [3.1] | 0.47 [0.19] vs. 0.49 [0.17] | 5.5 [3.3] vs. 5.8 [2.3] | 0.69 [1.1] vs. 0.60 [1.5] |
|  |  |  |  |  |  |
| **Clinical presentation** |  |  |  |  |  |
| (SA/UA+NSTEMI) | 5.8 [2.9] vs. 6.2 [5.6] | 6.1 [2.7] vs. 6.1 [4.0] | 0.47 [0.17] vs. 0.53 [0.25] | 5.8 [2.7] vs. 5.0 [2.1] | 0.67 [1.2] vs. 0.61 [1.1] |
|  |  |  |  |  |  |
| **Angiographic parameters** |  |  |  |  |  |
| No of vessels (single/multi) | 5.8 [3.1] vs. 5.9 [2.7] | 6.2 [3.2] vs. 5.7 [2.9] | 0.47 [0.16] vs. 0.49 [0.21] | 5.4 [2.9] vs. 5.9 [2.5] | 0.65 [1.1] vs. 0.63 [1.1] |
| Degree stenosis (<90%/>90%) | 5.8 [2.8] vs. 6.1 [2.2] | 6.0 [2.6] vs. 6.5 [2.6] | 0.52 [0.16] vs. 0.48 [0.18] | 5.8 [2.9] vs. 5.8 [1.9] | 0.61 [1.2] vs. 0.67 [1.2] |
| Occlusion (n/y) | 5.7 [2.9] vs. 6.2 [3.0] | 6.0 [2.8] vs. 6.2 [3.2] | 0.47 [0.16] vs. 0.49 [0.16] | 5.5 [2.9] vs. 5.8 [2.2] | 0.70 [1.2] vs. 0.59 [1.0] |
|  |  |  |  |  |  |
| **Event score** |  |  |  |  |  |
| (no/primary endpoint) | 5.8 [3.0] vs. 5.1 [1.9] | 6.1 [2.3] vs. 5.8 [3.2] | 0.47 [0.19] vs. 0.49 [0.14] | 6.0 [3.2] vs. 5.2 [1.9] | 0.68 [0.8] vs. 0.47 [1.4] |

**Table S1B. L-selectin expression**

BMI, body mass index; eGFR, estimated glomerular filtration rate; SA, stable angina pectoris; UA, unstable angina pectoris; NSTEMI, non-ST-elevated myocardial infarction, Previous coronary event = previous PCI and previous MI combined.
