# Supplementary material for: Toll-Like Receptor Induced CD11b and L-Selectin Response in Patients with Coronary Artery Disease
Source: PLoS One. 2013 Apr 3;8(4):e60467. doi: 10.1371/journal.pone.0060467 (PMC3616095; doi:10.1371/journal.pone.0060467)
Supplement: Table S2 — Dose-response curves and clinical characteristics. Dose-response curves of CD11b (A) and L-selectin (B) in relation to baseline clinical characteristics. For the multivariate analyses a backward linear regression model including age, gender, smoking, diabetes, hypertension, dyslipidemia, BMI, eGFR, previous coronary event, clinical presentation, number of diseased vessels, degree of stenosis, occlusion was used. Data are presented as median [IQR]. ∧p<0.05 in univariate analysis, *p<0.05 in multivariate analysis. (DOCX) [file pone.0060467.s002.docx]

**Table S2A. Dose-response CD11b after LPS stimulation**

|  | **Hillslope** | **EC50** | **AUC** | **Delta** |
| --- | --- | --- | --- | --- |
|  | **N=107** | **N=107** | **N=107** | **N=107** |
| **Risk Factors** |  |  |  |  |
| Age (<60/>60) | 0.70 [0.38] vs. 0.83 [0.65]^ | 0.03 [0.05] vs. 0.03 [0.05] | 39.6 [18.6] vs. 35.4 [10.9] | 17.2 [8.2] vs. 15.7 [6.6] |
| Gender (m/f) | 0.76 [0.41] vs. 0.93 [1.45]^* | 0.03 [0.05] vs. 0.02 [0.05] | 36.9 [15.5] vs. 37.5 [22.5] | 16.5 [6.6] vs. 15.4 [7.8] |
| Current smoker (n/y) | 0.81 [0.51] vs. 0.74 [0.42] | 0.03 [0.05] vs. 0.03 [0.03] | 37.0 [15.8] vs. 35.1 [16.6] | 16.1 [7.0] vs. 14.5 [7.2] |
| Diabetes (n/y) | 0.74 [0.41] vs. 0.98 [0.81] | 0.03 [0.05] vs. 0.02 [0.02] | 36.9 [16.3] vs. 37.0 [13.4] | 16.4 [7.3] vs. 15.7 [5.3] |
| Hypertension (n/y) | 0.79 [0.52] vs. 0.78 [0.45] | 0.04 [0.04] vs. 0.03 [0.05] | 37.2 [17.3] vs. 35.8 [14.6] | 16.6 [5.8] vs. 15.7 [7.4] |
| Dyslipidemia (n/y) | 0.74 [0.46] vs. 0.82 [0.50] | 0.03 [0.04] vs. 0.03 [0.04] | 38.4 [15.7] vs. 34.8 [16.3] | 16.6 [7.6] vs. 15.7 [7.1] |
| BMI (<25/>25) | 0.83 [0.52] vs. 0.75 [0.47] | 0.03 [0.05] vs. 0.03 [0.05] | 36.9 [18.9] vs. 37.2 [14.5] | 15.5 [6.8] vs. 16.3 [6.6] |
| eGFR (<60/>60) | 0.71 [0.67] vs. 0.80 [0.45] | 0.03 [0.05] vs. 0.03 [0.04] | 36.4 [18.5] vs. 37.0 [15.9] | 15.6 [8.0] vs. 16.6 [7.1] |
| Previous coronary event (n/y) | 0.75 [0.44] vs. 0.81 [0.61] | 0.03 [0.07] vs. 0.03 [0.04] | 35.4 [13.9] vs. 37.3 [21.1] | 15.8 [6.2] vs. 16.7 [8.2] |
|  |  |  |  |  |
| **Clinical presentation** |  |  |  |  |
| (SA/UA+NSTEMI) | 0.81 [0.53] vs. 0.69 [0.39] | 0.03 [0.04] vs. 0.02 [0.03] | 36.8 [16.5] vs. 39.6 [10.3] | 16.0 [7.2] vs. 16.6 [5.9] |
|  |  |  |  |  |
| **Angiographic parameters** |  |  |  |  |
| No of vessels (single/multi) | 0.81 [0.46] vs. 0.77 [0.45] | 0.03 [0.05] vs. 0.03 [0.04] | 38.2 [16.3] vs. 35.4 [12.8] | 17.0 [6.7] vs. 15.7 [7.8] |
| Degree stenosis (<90%/>90%) | 0.83 [0.69] vs. 0.74 [0.45] | 0.03 [0.06] vs. 0.03 [0.03] | 37.8 [16.0] vs. 34.5 [13.1] | 16.2 [7.0] vs. 15.3 [5.8] |
| Occlusion (n/y) | 0.82 [0.60] vs. 0.73 [0.26] | 0.03 [0.05] vs. 0.03 [0.02] | 36.9 [18.4] vs. 36.9 [12.1] | 16.3 [7.3] vs. 15.7 [6.3] |
|  |  |  |  |  |
| **Event score** |  |  |  |  |
| (no/primary endpoint) | 0.78 [0.48] vs. 0.81 [0.38] | 0.02 [0.05] vs. 0.03 [0.03] | 37.3 [16.2] vs. 39.9 [13.8] | 15.7 [7.2] vs. 16.6 [8.0] |

BMI, body mass index; eGFR, estimated glomerular filtration rate; SA, stable angina pectoris; UA, unstable angina pectoris; NSTEMI, non-ST-elevated myocardial infarction, Previous coronary event = previous PCI and previous MI combined**.**

|  | **Hillslope** | **EC50** | **AUC** | **Delta** |
| --- | --- | --- | --- | --- |
|  | **N=119** | **N=119** | **N=119** | **N=119** |
| **Risk Factors** |  |  |  |  |
| Age (<60/>60) | 1.56 [1.12] vs. 1.35 [1.03]* | 0.11 [0.10] vs. 0.12 [0.17] | 10.9 [4.2] vs. 11.8 [7.6]* | 5.6 [2.7] vs. 6.1 [2.6] |
| Gender (m/f) | 1.39 [0.99] vs. 1.59 [1.36] | 0.11 [0.13] vs. 0.12 [0.21] | 11.5 [5.2] vs. 9.5 [5.4] | 6.0 [2.7] vs. 5.8 [2.7] |
| Current smoker (n/y) | 1.44 [1.04] vs. 1.23 [0.64] | 0.11 [0.14] vs. 0.11 [0.18] | 11.5 [5.8] vs. 10.6 [4.9] | 6.0 [2.9] vs. 5.7 [2.3] |
| Diabetes (n/y) | 1.47 [0.94] vs. 1.19 [1.21] | 0.11 [0.13] vs. 0.09 [0.21] | 11.4 [4.6] vs. 11.1 [9.0] | 5.9 [2.3] vs. 6.6 [3.6] |
| Hypertension (n/y) | 1.41 [1.05] vs. 1.39 [0.93] | 0.09 [0.10] vs. 0.14 [0.15]^ | 12.3 [7.5] vs. 10.9 [4.9]^* | 6.1 [3.4] vs. 5.8 [2.6] |
| Dyslipidemia (n/y) | 1.34 [0.83] vs. 1.59 [1.13]^ | 0.12 [0.16] vs. 0.11 [0.12] | 11.4 [6.5] vs. 11.3 [4.8] | 6.0 [2.8] vs. 5.9 [2.8] |
| BMI (<25/>25) | 1.35 [0.94] vs. 1.55 [1.07] | 0.12 [0.14] vs. 0.10 [0.12] | 10.6 [7.3] vs. 11.4 [4.2] | 6.0 [3.3] vs. 5.8 [2.3] |
| eGFR (<60/>60) | 1.53 [5.74] vs. 1.39 [1.00]* | 0.16 [0.24] vs. 0.11 [0.12]* | 10.8 [7.2] vs. 11.5 [5.4] | 6.7 [3.4] vs. 5.9 [2.7] |
| Previous coronary event (n/y) | 1.55 [1.32] vs. 1.35 [0.91] | 0.10 [0.13] vs. 0.12 [0.15] | 10.9 [5.6] vs. 11.4 [5.5] | 5.8 [3.0] vs. 6.0 [2.5] |
|  |  |  |  |  |
| **Clinical presentation** |  |  |  |  |
| (SA/UA+NSTEMI) | 1.54 [0.96] vs. 1.17 [0.92] | 0.11 [0.14] vs. 0.11 [0.18] | 11.1 [4.9] vs. 13.2 [5.9] | 5.8 [2.6] vs. 6.4 [5.2] |
|  |  |  |  |  |
| **Angiographic parameters** |  |  |  |  |
| No of vessels (single/multi) | 1.47 [0.84] vs. 1.35 [1.23] | 0.13 [0.12] vs. 0.11 [0.15] | 11.2 [4.6] vs. 11.5 [6.0] | 6.0 [2.5] vs. 5.9 [2.6] |
| Degree stenosis (<90%/>90%) | 1.67 [1.48] vs. 1.35 [1.01]^ | 0.12 [0.17] vs. 0.10 [0.14] | 10.3 [5.4] vs. 12.4 [6.4]^ | 5.6 [2.6] vs. 6.6 [2.5]^ |
| Occlusion (n/y) | 1.58 [0.95] vs. 1.21 [0.93]^ | 0.11 [0.14] vs. 0.11 [0.12] | 11.1 [5.0] vs. 11.7 [7.6] | 5.7 [2.6] vs. 6.4 [3.2] |
|  |  |  |  |  |
| **Event score** |  |  |  |  |
| (no/primary endpoint) | 1.35 [1.04] vs. 1.55 [1.32] | 0.11 [0.13] vs. 0.14 [0.16] | 11.5 [4.9] vs. 11.0 [4.3] | 6.0 [2.7] vs. 5.2 [2.2] |

**Table S2B. Dose-response L-selectin after LPS stimulation**

BMI, body mass index; eGFR, estimated glomerular filtration rate; SA, stable angina pectoris; UA, unstable angina pectoris; NSTEMI, non-ST-elevated myocardial infarction, Previous coronary event = previous PCI and previous MI combined**.**
